# Primary diffuse large b-cell lymphoma of the lip: a case report and literature review

**DOI:** 10.3389/fonc.2026.1763767

**Published:** 2026-06-08

**Authors:** Pingping Liu, Changjiang Xu, Liming Yu, Xiuying Wang, Yan Wang, Xiaofei Yan

**Affiliations:** 1 Department of Ultrasound, Shengli Oilfield Central Hospital, Shandong, China; 2 Department of Gastroenterology, Shengli Oilfield Central Hospital, Shandong, China; 3 Department of Spinal Surgery, Shengli Oilfield Central Hospital, Shandong, China; 4 Department of Oncology, Shengli Oilfield Central Hospital, Shandong, China

**Keywords:** DLBCL, germinal center B-cell, immunohistochemistry, lip, misdiagnosis, multidisciplinary approach, primary

## Abstract

**Background:**

Primary diffuse large B-cell lymphoma (DLBCL) of the lip is a rare subtype of extranodal non-Hodgkin lymphoma that presents a diagnostic challenge due to its infrequent location and nonspecific clinical symptoms. The early suspicion of this diagnosis is often delayed because of its overlap with benign inflammation/infection, such as abscesses and mucoceles.

**Case presentation:**

This report describes a 65-year-old male with worsening swelling of the upper lip that was initially diagnosed as a maxillary space infection. Imaging studies, including ultrasound and MRI, showed a poorly defined mass, and an axial biopsy with immunohistochemistry confirmed a diagnosis of the germinal center B-cell (GCB) subtype of DLBCL. The patient was ultimately treated successfully with R-CHOP chemotherapy and achieved complete remission.

**Conclusion:**

This case illustrates both the importance of considering lymphoma in the differential diagnosis of a lip lesion that has persisted and requires ongoing clinical evaluation, as well as the benefit of multidisciplinary care for appropriate diagnosis and timely management.

## 1 Introduction

Diffuse large B-cell lymphoma (DLBCL) is the most common and aggressive subtype of non-Hodgkin lymphoma (NHL), accounting for approximately 30%–40% of adult lymphoma cases (1). While extranodal involvement is frequent, primary DLBCL of the lip is exceptionally rare, comprising less than 1% of all extranodal DLBCL cases (2, 3). This rarity is compounded by the lip’s typical absence of lymphoid tissue and its unique anatomical environment, which includes abundant mucosal tissue, minor salivary glands, and small vessels.

Clinically, lip DLBCL often presents with nonspecific signs such as swelling and tenderness, overlapping with benign conditions like infections, mucous cysts, or vascular lesions (3, 4). This frequently leads to initial misdiagnosis, as exemplified in the current case where the patient was initially treated for a maxillary space infection. Such diagnostic delays underscore the importance of maintaining a high index of suspicion for malignancy in persistent or atypical lip swellings.

Here we report a case of primary DLBCL of the upper lip in a 65-year-old male, detailing the clinical presentation, diagnostic workup, treatment response, and relevant literature. This case highlights the diagnostic and therapeutic challenges posed by rare malignancies in uncommon sites and emphasizes the need for a multidisciplinary approach to ensure timely diagnosis and appropriate management.

## 2 Case presentation

An individual aged 65 visited our clinic with a 20-day history of worsening swelling in his upper lip. The mild swelling had progressed in the last four days, whereby he was compelled to seek medical attention. He complained of discomfort associated with tightness of the skin and tenderness to palpation overlying the swollen area, but reported he had no difficulty swallowing, fever, or any systemic symptoms such as weight loss or night sweats. On examination, the upper lip was swollen, and there was a firm, tender mass measuring approximately 4.5×3.2cm. The skin overlying the mass was tight, and there was a mild restriction of mouth opening. There were no abnormal findings in the oral cavity or on examination of the surrounding facial structures.

The patient had been to another hospital for treatment, where he was diagnosed with a “maxillary space infection.” He was placed on antibiotics, but did not experience improvement in his symptoms. Due to a lack of improvement in his clinical symptoms, he was referred to us for further assessment. The patient’s clinical history obtained upon admission was otherwise unremarkable, with no significant past medical history. There was no past medical history of cardiovascular, respiratory, or metabolic diseases, there was no reported recent history of trauma, and there were no known allergies or recent exposure to infectious agents. His personal medical history was negative, and his family medical history was unremarkable. The baseline characteristics of this patient are outlined in **Table 1**.

**Table 1:** Baseline characteristics of the patient.

Characteristic	Details
Age	65 years
Gender	Male
Presenting Symptoms	Progressive swelling of the upper lip for 20 days, worsened over the last 4 days
Physical Examination Findings	Swollen, tender upper lip (4.5 x 3.2 cm), tight skin, mild restriction in mouth opening, no fever or swallowing difficulties
Medical History	No significant medical history (no cardiovascular, metabolic, or respiratory diseases)
Previous Treatment	Initially misdiagnosed as a maxillary space infection, treated with antibiotics, and no improvement
Imaging Studies	Ultrasound: Hypoechoic mass, irregular borders; MRI: Irregular, slightly hyperintense mass
Pathology	Histopathology: Axial biopsy showing diffuse large B-cells with starry-sky pattern and necrosis; Immunohistochemistry: CD20+, CD79a+, CD10+, BCL-6+, MUM1+ (GCB subtype)
Molecular Testing	No MYC/BCL2 rearrangements, no EBV infection

**Figure 1:**
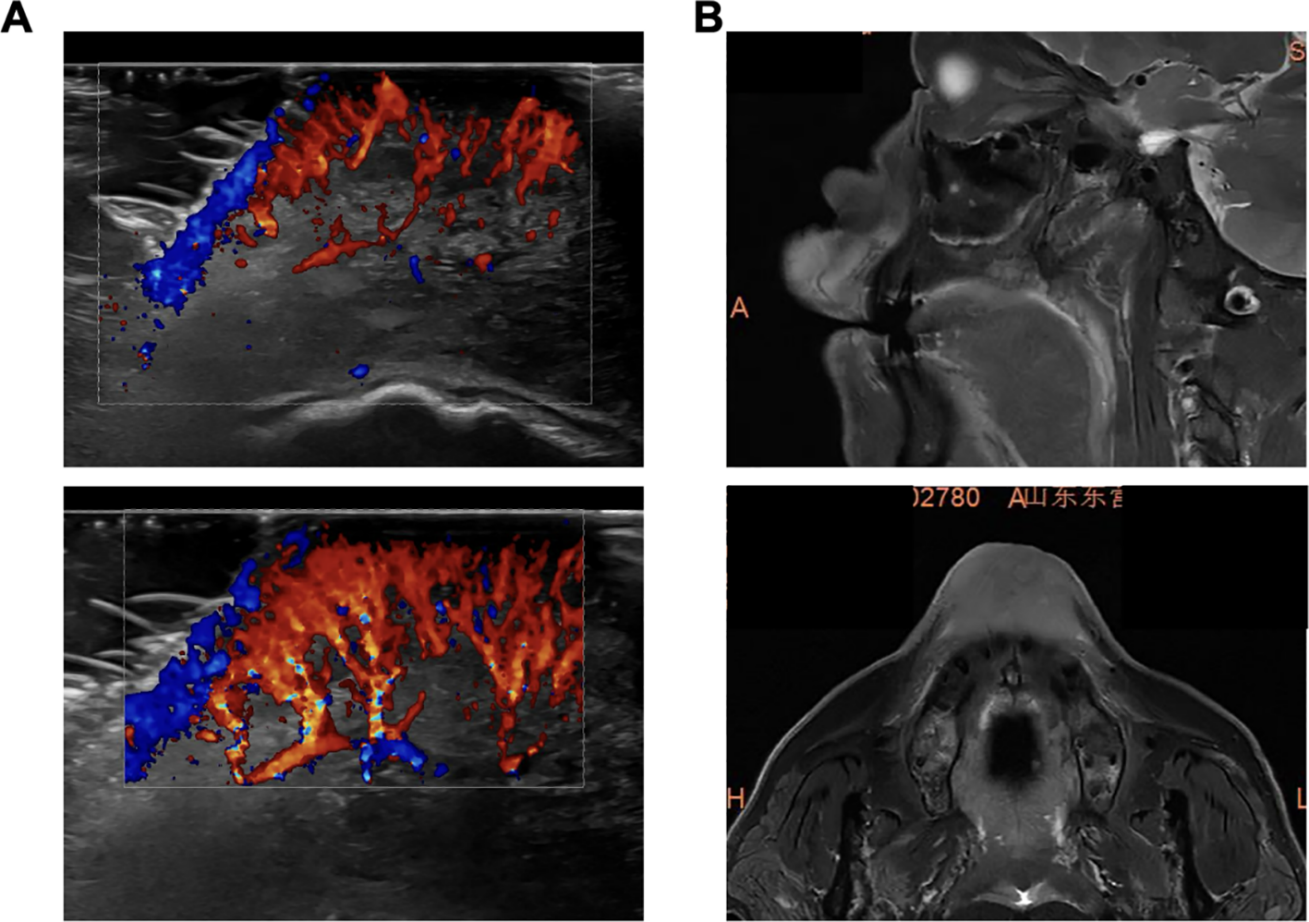
Representative images of ultrasound and MRI examinations. **(A)** Ultrasound examination revealed a hypoechoic mass with irregular borders and no clear demarcation from surrounding tissues. **(B)** MRI imaging showed an ill-defined lesion with a slight enhancement.

**Figure 2:**
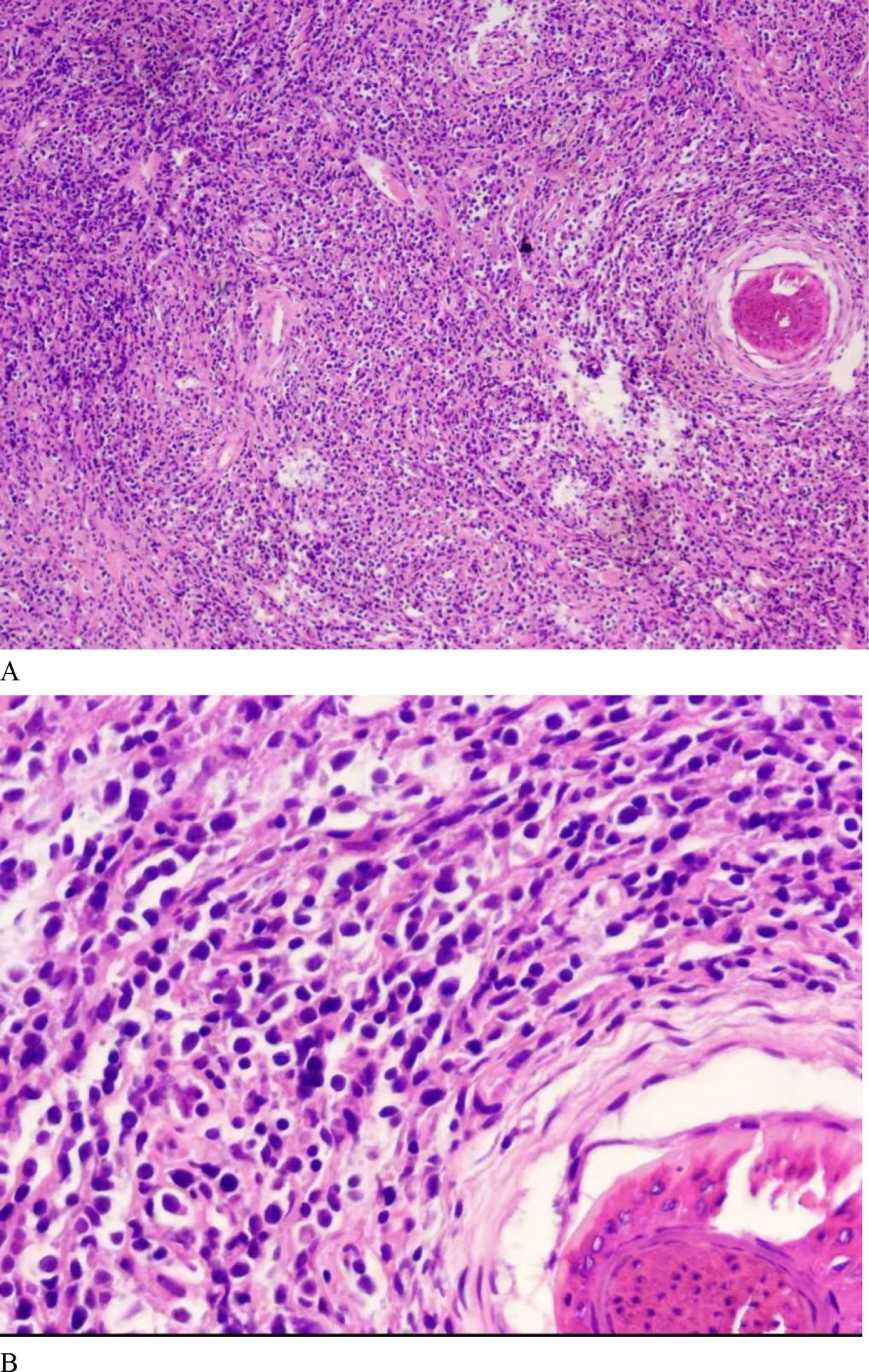
Histological examination of primary diffuse large B-cell lymphoma (DLBCL) in lip tissue. **(A)** A low magnification view (×100) showing the general architecture of the lip tissue involved with diffuse large B-cell lymphoma, characterized by a mixture of atypical lymphoid cells and necrotic areas. **(B)** A higher magnification view (×400) revealing large, atypical lymphoid cells with irregular nuclei and prominent nucleoli, typical of diffuse large B-cell lymphoma.

**Figure 3:**
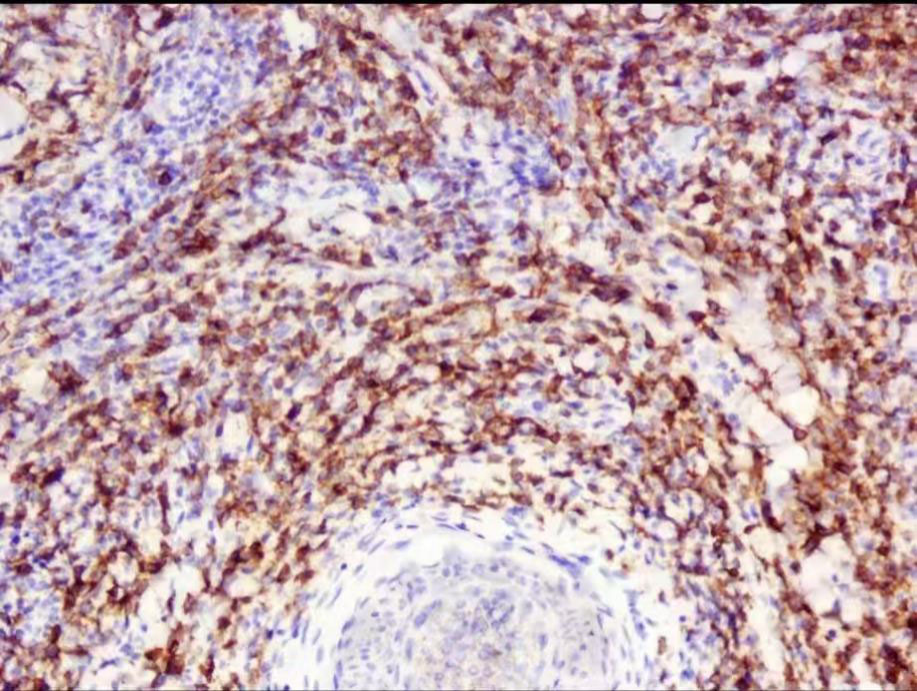
Immunohistochemical staining (CD20) of primary diffuse large B-cell lymphoma (DLBCL) in lip tissue. This image shows a high magnification (×400) of lip tissue stained for B-cells. The brown staining highlights the presence of CD20-positive atypical lymphoid cells, confirming the diagnosis of B-cell lymphoma.

To characterize the lesion further, imaging studies were performed. Ultrasound examination showed a hypoechoic mass with irregular borders and no clear demarcation from the surrounding tissues (**Figure 1A**); MRI imaging demonstrated an ill-defined lesion, which exhibited only mild enhancement, suggestive of a malignant process (**Figure 1B**). Histopathological examination of the biopsy specimen revealed a dense infiltration of atypical lymphoid cells. At low magnification, the tissue architecture showed diffuse growth with areas of necrosis (**Figure 2A**). High magnification further identified these cells as large atypical lymphoid cells characterized by irregular nuclei and prominent nucleoli, which are hallmark features of DLBCL (**Figure 2B**). Following the histopathological review, immunohistochemical (IHC) staining was performed to further characterize the lesion. The atypical lymphoid cells demonstrated positivity for CD20, CD79a, and CD10, as well as BCL-6 and MUM1 (**Figure 3**), confirming the diagnosis of B-cell lymphoma. According to the Hans algorithm, the tumor was classified as the germinal center B-cell (GCB) subtype due to CD10 positivity. In the Hans algorithm, CD10 expression is the primary determinant for the GCB subtype; if the atypical lymphoid cell express CD10 (>30%), it is classified as GCB regardless of BCL-6 or MUM1 status. Molecular testing excluded EBV infection, and MYC/BCL2 rearrangements further supported the diagnosis of DLBCL.

The patient underwent routine hematological investigations, which showed mild anemia (hemoglobin 11.8g/dl), normal leukocytes, and normal lactate dehydrogenase (LDH); findings that were tenuous in ruling in or out the presence of lymphoma. Serum biochemistry and inflammatory markers were all normal and did not help with the case-specific diagnosis.

The patient was treated with six cycles of standard R-CHOP (rituximab, cyclophosphamide, doxorubicin, vincristine, and prednisone) chemotherapy. A PET-CT scan completed six months after treatment demonstrated complete metabolic remission. The patient remained disease-free at the year follow-up visit with no clinical or radiological evidence of recurrence.

## 3 Discussion and literature review

Primary DLBCL of the lip represents a very rare manifestation of malignancy. The diagnosis of this condition is further complicated by the anatomical features of the lip itself, as there is little lymphoid tissue present in the lip compared to other sites in the body. The lip is comprised primarily of mucosal and soft tissue and can be confused with benign presentations, since conditions such as infections, mucous cysts, and vascular lesions may demonstrate similar signs, such as swelling and tenderness (8, 9). In our instance, the absence of systemic “B symptoms” and the misleading infectious presentation likely delayed the diagnosis, which is consistent with previous reports around extranodal DLBCL of the lip (5, 6). This indicates that atypical presenting complaints that may resemble odontogenic or soft tissue infections may contribute to delays in diagnosis, and ultimately suggests that a high index of suspicion be maintained, especially in older patients with ongoing lip lesions.

Imaging, such as ultrasound or MRI, plays a supportive role in identifying the anatomical extent and suggesting malignancy, though definitive diagnosis and subtyping of lip DLBCL require histopathological and immunohistochemical evaluation. In this case, ultrasound demonstrated a hypoechoic, ill-defined mass, which is often seen with malignancy (7, 8). MRI further characterized the lesion by identifying the depth of the lesion, but also increased suspicion for malignancy due to the appearance of irregular, infiltrative borders. Imaging in this case was very useful during the biopsy and for monitoring the treatment response (**Figure 1**). Nevertheless, as we have illustrated in this case, while imaging allows us to ascertain the degree of the ailment, histopathologic assessment via axial biopsy and immunohistochemical analysis provide the definitive diagnosis of the particular DLBCL.

Primary DLBCL of the lip is an extremely rare process, with only a limited number of confirmed cases documented in the medical literature (9–11). Representative reports include Do Nascimento et al. (2020), describing a case in the upper lip of a 73-year-old woman (9), as well as recent publications by Seki et al. (2024) and Issa et al. (2024), which added a confirmed case and a review of similar presentations, respectively (10, 11). A summary of previously reported cases is provided in **Table 2**, illustrating the clinical and demographic features of this rare entity. As illustrated in **Table 2**, primary DLBCL of the lip exhibits heterogeneity in its molecular subtypes. While earlier reports, such as Do Nascimento et al. (2020), and our present case involve the GCB subtype, recent cases by Seki et al. (2024) and Issa et al. (2024) have identified the non-GCB (ABC) subtype in this anatomical site (9–11). While the large majority of extra-nodal DLBCL cases occur in more common sites such as the gastrointestinal tract or central nervous system, the lip’s unique anatomical environment—lacking significant lymphoid tissue—presents a distinct diagnostic challenge. The large majority of extra-nodal DLBCL cases describe more common sites, such as the GI tract, skin, or central nervous system (12–16). Due to the extreme rarity of primary lip DLBCL, there is currently limited clinical evidence to establish standardized treatment protocols specifically for this anatomical site. Several reports indicate that management for lip DLBCL should be made on an individualized basis, depending on the location of the lesion, size of the tumor, and overall health of the patient (17, 18). For primary DLBCL of the localized lip, the preferred management is usually chemotherapy plus localized radiation therapy, as this may often provide adequate, yet less severe oncologic treatment while keeping the extra lip functional and cosmetically intact.

**Table 2:** The previously reported cases of lip DLBCL.

Author(s)	Year	Age	Gender	Clinical presentation	Subtype (Hans)	Treatment	Outcome
Seki et al. (10)	2024	63	Female	Swelling and tenderness of upper lip	non-GCB	R-CHOP chemotherapy	Remission
Issa et al. (11)	2024	70	Male	Firm mass on lip, misdiagnosed as infection	non-GCB	Chemotherapy	Disease-free at follow-up
Do Nascimento GJF et al. (9)	2020	73	Female	Upper lip	GCB	Chemotherapy	Death
Present Case	2026	65	Male	Swelling of upper lip, misdiagnosed as infection	GCB	R-CHOP chemotherapy	Complete remission

For the treatment, R-CHOP (rituximab, cyclophosphamide, doxorubicin, vincristine, and prednisone) is the preferred regimen for therapy of DLBCL, including extra-nodal cases (19, 20). R-CHOP has proved effective and has been shown to control disease and improve survival outcomes. In the current case, the patient’s achievement of complete remission follows the successful therapeutic trends observed in recent literature (**Table 2**). This reinforces the effectiveness of R-CHOP in rare extra-nodal sites and highlights that timely initiation of immunochemotherapy can lead to sustained remission, particularly in the GCB subtype. Patients who do present as high-risk, such as non-GCB or elevated Ki-67, may require more aggressive treatment options that may include CNS withdrawal (21).

The prognosis in DLBCL is dependent on varied factors, which include not only the subtype of the lymphoma but also the International Prognostic Index (IPI) score (22) and whether the genetic mutations are prevalent as a high-risk factor. The identification of the GCB subtype in this case is clinically significant. Although both GCB and non-GCB subtypes reported in primary lip DLBCL can achieve remission with prompt immunochemotherapy, the GCB subtype is generally associated with a more favorable long-term prognosis, consistent with our patient’s rapid and complete metabolic response (1, 3). The patient’s robust response suggests that early multidisciplinary involvement and the prompt administration of R-CHOP treatment can significantly improve the clinical trajectory, even in rare extranodal presentations. The inclusion of targeted therapy, such as BCL-2 inhibitors or novel treatments like CAR-T therapy, may further improve the next case outcomes (23, 24). However, more evidence is required to establish the role of these targeted agents in improving outcomes of DLBCL in rare extra-nodal cases, like the lip.

## 4 Conclusion

Primary DLBCL of the lip is an exceptionally rare malignancy, often misdiagnosed due to its atypical presentation and nonspecific clinical features. This case highlights the importance of considering lymphoma in the differential diagnosis of unexplained lip masses, particularly in older patients without systemic B-symptoms. While imaging studies aid in determining the anatomical extent, definitive diagnosis depends on histopathological assessment via axial biopsy and immunohistochemical analysis, which confirmed the GCB subtype in this patient. Consistent with the typically favorable prognosis of the GCB subtype, the patient achieved complete metabolic remission with standard R-CHOP chemotherapy, further demonstrating the potential for excellent outcomes with timely intervention. Early recognition of this rare entity and a multidisciplinary diagnostic approach can significantly improve patient outcomes. Further accumulation of similar case reports and clinical data will be crucial to refine treatment protocols and enhancing understanding of this uncommon presentation.

## Data Availability

The original contributions presented in the study are included in the article/supplementary material. Further inquiries can be directed to the corresponding author.
