# Correction: Primary diffuse large B-cell lymphoma of the lip: a case report and literature review

**DOI:** 10.3389/fonc.2026.1903276

**Published:** 2026-06-16

**Authors:** Pingping Liu, Changjiang Xu, Liming Yu, Xiuying Wang, Yan Wang, Xiaofei Yan

**Affiliations:** 1 Department of Ultrasound, Shengli Oilfield Central Hospital, Shandong, China; 2 Department of Gastroenterology, Shengli Oilfield Central Hospital, Shandong, China; 3 Department of Spinal Surgery, Shengli Oilfield Central Hospital, Shandong, China; 4 Department of Oncology, Shengli Oilfield Central Hospital, Shandong, China

**Keywords:** Germinal center B-cell, Immunohistochemistry, Misdiagnosis, multidisciplinary approach, Primary lip DLBCL

Author Yan Wang was erroneously assigned as corresponding author. The correct corresponding author is Pingping Liu.

Author Pingping Liu, Changjiang Xu, Liming Yu were erroneously omitted as equal contributing author.

The original version of this article has been updated.

